# Defects in the Peripheral Taste Structure and Function in the MRL/lpr Mouse Model of Autoimmune Disease

**DOI:** 10.1371/journal.pone.0035588

**Published:** 2012-04-19

**Authors:** Agnes Kim, Pu Feng, Tadahiro Ohkuri, Daniel Sauers, Zachary J. Cohn, Jinghua Chai, Theodore Nelson, Alexander A. Bachmanov, Liquan Huang, Hong Wang

**Affiliations:** Monell Chemical Senses Center, Philadelphia, Pennsylvania, United States of America; German Institute for Human Nutrition, Germany

## Abstract

While our understanding of the molecular and cellular aspects of taste reception and signaling continues to improve, the aberrations in these processes that lead to taste dysfunction remain largely unexplored. Abnormalities in taste can develop in a variety of diseases, including infections and autoimmune disorders. In this study, we used a mouse model of autoimmune disease to investigate the underlying mechanisms of taste disorders. MRL/MpJ-Fas^lpr^/J (MRL/lpr) mice develop a systemic autoimmunity with phenotypic similarities to human systemic lupus erythematosus and Sjögren's syndrome. Our results show that the taste tissues of MRL/lpr mice exhibit characteristics of inflammation, including infiltration of T lymphocytes and elevated levels of some inflammatory cytokines. Histological studies reveal that the taste buds of MRL/lpr mice are smaller than those of wild-type congenic control (MRL/+/+) mice. 5-Bromo-2′-deoxyuridine (BrdU) pulse-chase experiments show that fewer BrdU-labeled cells enter the taste buds of MRL/lpr mice, suggesting an inhibition of taste cell renewal. Real-time RT-PCR analyses show that mRNA levels of several type II taste cell markers are lower in MRL/lpr mice. Immunohistochemical analyses confirm a significant reduction in the number of gustducin-positive taste receptor cells in the taste buds of MRL/lpr mice. Furthermore, MRL/lpr mice exhibit reduced gustatory nerve responses to the bitter compound quinine and the sweet compound saccharin and reduced behavioral responses to bitter, sweet, and umami taste substances compared with controls. In contrast, their responses to salty and sour compounds are comparable to those of control mice in both nerve recording and behavioral experiments. Together, our results suggest that type II taste receptor cells, which are essential for bitter, sweet, and umami taste reception and signaling, are selectively affected in MRL/lpr mice, a model for autoimmune disease with chronic inflammation.

## Introduction

Taste disorders, including taste loss and taste distortion, often occur concomitantly with various diseases. Taste loss is the decreased ability or inability to detect or recognize taste compounds. Taste distortion refers to altered taste quality perception or phantom taste [1], [2]. In some patient populations, taste dysfunction contributes significantly to anorexia, malnutrition, and poor general health [3], [4], [5], [6]. Because little is known of the molecular etiology of taste abnormalities, treatment options for such taste disorders are very limited [7], [8].

Several autoimmune diseases are known to affect taste function [2], [9]. Sjögren's syndrome is a systemic autoimmune disorder characterized by the infiltration of immune cells into salivary and lacrimal glands [10], [11]. Sjögren's syndrome patients show decreased taste sensitivity for bitter, sour, salty, and sweet compounds [12], [13], [14]. Most Sjögren's syndrome patients experience reduced production of saliva, with accompanying dryness of the mouth. While proposed as a contributing factor in decreased taste sensitivity [13], low saliva production does not always show a direct correlation with taste loss in these patients [12], [14], [15]. Taste alterations also occur in other autoimmune diseases, such as systemic lupus erythematosus and insulin-dependent diabetes mellitus [2], [9], but the taste impairment in these diseases has not been well characterized.

The peripheral transduction mechanisms of taste signals are becoming clearer, providing us with a comparative basis for describing alterations in taste dysfunction. Taste compounds are detected in the mouth by cells in taste buds [16], [17]. Although consisting of only 50–100 cells, the taste bud is a complex structure with specialized sensory cells performing different, but integrated, functions. Recent studies generally support the notion that different taste qualities are recognized by distinct subsets of taste cells [18].

Taste bud cells undergo constant turnover, with an average life span of about 10 days [19], [20]. The mechanisms that regulate the life span and turnover of taste cells have not been fully determined [21]. Our recent studies shed light on this process by showing that normal taste buds express various genes involved in immune responses [22], [23], [24], and that inflammatory stimuli, such as lipopolysaccharide (LPS), can distort taste cell turnover and taste progenitor cell proliferation.

In this study, we investigated the mechanisms of taste disorders using the mouse MRL/MpJ-Fas^lpr^/J (MRL/lpr) autoimmune disease model. Our results suggest that taste bud cell renewal is inhibited in these mice. In addition, molecular, cellular, and functional analyses indicate that type II taste receptor cells, which are responsible for bitter, sweet, and umami taste reception and signaling, are selectively affected in this disease model.

## Materials and Methods

### Ethics Statement

This study was performed in strict accordance with the recommendations in the Guide for the Care and Use of Laboratory Animals of the National Institutes of Health. All experiments involving animals were performed according to the protocol approved by the Monell Chemical Senses Center Institutional Animal Care and Use Committee (approved protocol # no. 1122). All surgery was performed under sodium pentobarbital anesthesia, and every effort was made to minimize suffering.

### Animals

MRL/MpJ-Fas^lpr^/J (MRL/lpr) mice and their wild-type congenic control MRL/MpJ (MRL/+/+) mice were purchased from the Jackson Laboratory (Bar Harbor, ME) and were bred and housed in a climate-controlled environment at the animal care facility of the Monell Chemical Senses Center. MRL/lpr mice carry a spontaneous mutation in the *Fas* antigen gene resulting from integration of an endogenous retroviral sequence [25], [26]. This mutation abolishes the expression and function of *Fas* and results in autoantibody production and the accumulation of large numbers of T cells in lymph nodes and spleen. MRL/lpr mice develop an autoimmune disease that resembles human systemic lupus erythematosus and Sjögren's syndrome. This mouse strain has been widely used as an animal model for these human diseases [27], [28]. Symptoms of autoimmune disease appear at about 2–3 months of age and gradually progress in these mice. Both female and male MRL/lpr mice were included in this study. Sex- and age-matched MRL/+/+ mice were used as controls.

### Reagents

5-Bromo-2′-deoxyuridine (BrdU) and all taste compounds were obtained from Sigma (St. Louis, MO), except hydrochloric acid (HCl) which was purchased from VWR Scientific (Radnor, PA). An affinity-purified rabbit polyclonal antibody against interferon-γ (IFN-γ), as well as control rabbit IgG, was purchased from PeproTech (Rocky Hill, NJ) [24]. Affinity-purified goat polyclonal antibodies against tumor necrosis factor-α (TNF-α) and carbonic anhydrase 4 (CA4) were purchased from R&D Systems (Minneapolis, MN) [24], [29]. Rabbit polyclonal antibodies against gustducin (sc-395) and phospholipase C-β2 (PLC-β2) (sc-206) were purchased from Santa Cruz Biotechnology (Santa Cruz, CA) [30], [31], [32], [33]. Rabbit polyclonal antibodies against neural cell adhesion molecule (NCAM) and synaptosomal-associated protein 25 (SNAP25) were purchased from Chemicon (Temecula, CA) and Sigma, respectively [34], [35]. Antibodies against Ki67 and CD3 were obtained from BD Biosciences (Pasadena, CA) [24]. A rabbit polyclonal antibody against KCNQ1 was purchased from Millipore (Billerica, MA) [36]. The anti-BrdU monoclonal antibody developed by S. J. Kaufman was obtained from the Developmental Studies Hybridoma Bank developed under the auspices of the National Institute of Child Health and Human Development and maintained by the University of Iowa Department of Biological Sciences (Iowa City, IA) [37]. Cyanine 3 (Cy3)-conjugated goat anti-rabbit antibody, DyLight-649-conjugated donkey anti-rabbit antibody, Cy5-conjugated donkey anti-goat antibody, and DyLight-488-conjugated donkey anti-goat antibody were purchased from Jackson ImmunoResearch Laboratories (West Grove, PA). The Zenon mouse and rabbit antibody labeling kits were purchased from Invitrogen (Carlsbad, CA). Collagenase A and dispase II were purchased from Roche Applied Sciences (Indianapolis, IN). Anti-Rat Ig HRP Detection Kit was purchased from BD Biosciences. Vectashield H-1000 mounting medium and H-1200 mounting medium containing 4′, 6-diamidino-2-phenylindole (DAPI) were purchased from Vector Laboratories (Burlingame, CA).

### Quantitative real-time RT-PCR analysis (qRT-PCR)

MRL/+/+ and MRL/lpr mice were euthanized, and tongue epithelial samples were prepared as previously described [22]. Total RNA was extracted using Absolutely RNA Microprep Kit (Stratagene, Cedar Creek, TX) from peeled-off epithelial pieces that either lacked taste buds (nontaste epithelium, excised from within the intermolar eminence) or contained foliate or circumvallate taste buds (taste epithelium). Tissues from 3–4 mice per group (MRL/lpr or MRL/+/+) and per sample type (nontaste or taste epithelium) were pooled for each sample set. Three independent sets of samples were prepared. Together, 9–12 mice from each group were included in the experiments. Approximately equal amounts of total RNA from these samples were reverse transcribed into cDNA using Superscript III reverse transcriptase (Invitrogen). Quantitative real-time PCR (qPCR) reactions were set up using Power SYBR Green PCR Master Mix (Applied Biosystems, Foster City, CA) in duplicate or triplicate and run on an ABI PRISM 7000 Sequence Detection System (Applied Biosystems). Relative quantification of gene expression was performed using the ABI software, which was based on the 2^−ΔΔCt^ method [38]. β-Actin was used as the endogenous control gene for these analyses. RT-PCR primers were designed to place the forward and the reverse primers in separate exons of each gene when more than one exon is present. The specificity of the PCR reactions was analyzed by dissociation studies using the ABI instrument and confirmed by agarose gel electrophoresis. RT-PCR primers are listed in Table 1.

**Table 1:** RT-PCR primers.

Gene name	GenBank accession #no.*	Forward primer	Reverse primer	Product (bp)**
**Cytokines:**				
IFN-γ	NM_008337	AGCAACAGCAAGGCGAAAA	CTGGACCTGTGGGTTGTTGA	71
IL-10	NM_010548	AAGGCAGTGGAGCAGGTGAA	CCAGCAGACTCAATACACAC	159
IL-6	NM_031168	TCATATCTTCAACCAAGAGGTA	CAGTGAGGAATGTCCACAAACTG	230
TNF-α	NM_013693	CCTCACACTCAGATCATCTTCTCA	TGGTTGTCTTTGAGATCCATGC	147
**Taste cell markers:**				
Gustducin	NT_165760	AAGTGATATCCTGGGCTTTAGA	GCTTTCAGTTTGAAAGGCATCA	198
TrpM5	NM_020277	CCCTGTCTTACCCTGAGTTC	GCATTGGGTACCCTTGAGCA	205
NeuroD	NM_010894	GAGATCGTCACTATTCAGAACC	TTCTTGTCTGCCTCGTGTTCC	161
SNAP25	NM_011428	TGGATGAACGGGAGCAGATG	GTTGCACGTTGGTTGGCTTC	238
PKD2L1	NM_181422	TTGTGTCCCAGATTGATGCT	CTTTTCCATCCTCCTGTCCA	172
**Endogenous control:**				
β-Actin	NM_007393	GATTACTGCTCTGGCTCCTA	ATCGTACTCCTGCTTGCTGA	142

* GenBank accession number used for primer design.

** Size of predicted PCR product in base pairs (bp).

### Immunohistochemistry

Excised mouse tongue tissues were fixed in freshly prepared 4% paraformaldehyde (PFA) in phosphate-buffered saline (PBS) for 1 h on ice and then cryoprotected in 20% sucrose/PBS solution at 4°C overnight. Tissues were embedded in Neg-50 mounting medium (Richard-Allan Scientific, Kalamazoo, MI) and sliced into 10- to 12-µm-thick sections using a Microm HM 500 OM cryostat (Thermo Scientific Microm, Walldorf, Germany). Sections were mounted on StarFrost adhesive slides (Mercedes Medical, Sarasota, FL). Circumvallate sections were cut in parallel to the surface of the tongue. About 26–28 taste-bud-containing serial sections were collected from each circumvallate papilla. Fungiform sections were collected from the tip of the tongue, which was cut coronally. For immunostaining of TNF-α and IFN-γ, tissue sections were washed three times with PBS containing 0.3% Triton X-100 and then incubated with a permeabilization buffer (0.1% saponin and 0.009% sodium azide) at room temperature for 1 h, followed by incubation with a blocking buffer (3% bovine serum albumin, 0.3% Triton X-100, 2% goat or horse serum, and 0.1% sodium azide in PBS) containing 0.1% saponin at room temperature for 1 h. The sections were then incubated with either an affinity-purified rabbit antibody against IFN-γ or an affinity-purified goat antibody against TNF-α in the blocking buffer at room temperature for 1 h or at 4°C overnight. The sections were washed and then incubated with a Cy3-conjugated goat anti-rabbit secondary antibody (for IFN-γ staining) or a DyLight-488-conjugated donkey anti-goat antibody (for TNF-α staining) at room temperature for 1 h. Sections were washed again for three times with PBS containing 0.3% Triton X-100. For colocalization studies of IFN-γ, rabbit polyclonal antibodies against PLC-β2 and SNAP25 were labeled using the Alexa488 Zenon rabbit IgG labeling kit following the manufacturer's recommended protocol. The freshly labeled antibodies were added to the slides within 30 min of preparation and incubated at room temperature for 1–2 h. The sections were washed twice with PBS containing 0.3% Triton X-100 solution and once with PBS (pH 7.4) and then postfixed in freshly prepared 4% PFA/PBS for 15 min at room temperature. Sections were washed again and mounted with Vectashield H-1000 mounting medium and imaged with a Leica TCS SP2 spectral confocal microscope (Leica Microsystems, Wetzlar, Germany). For colocalization studies of TNF-α, the rabbit polyclonal antibody against PLC-β2 was paired with DyLight-649-conjugated donkey anti-rabbit secondary antibody.

For immunostaining using anti-gustducin and anti-NCAM antibodies, processed tissue sections were incubated with rabbit polyclonal antibodies against these cell markers at 4°C overnight, followed by incubation with the Cy3-conjugated goat anti-rabbit secondary antibody. We chose four circumvallate sections out of 26–28 taste-bud-containing serial sections from each circumvallate papilla for anti-gustducin or anti-NCAM immunostaining. The four sections for each immunostaining were chosen from different positions along the circumvallate trench, and they were separated from each other by at least 40 µm (four 10-µm-thick sections away). The sections used for anti-gustducin and anti-NCAM immunostaining were adjacent sections. Five mice per group were included in the experiment.

For immunostaining using anti-gustducin and anti-CA4 antibodies, tissue sections containing fungiform papillae were collected from the tip of the tongue. Sections were incubated with a rabbit polyclonal antibody against gustducin and a goat polyclonal antibody against CA4 at 4°C overnight. A DyLight-649-conjugated donkey anti-rabbit antibody and a DyLight-488-conjugated donkey anti-goat antibody were then incubated with the tissue sections at room temperature for 1 h. Vectashield H-1200 mounting medium containing DAPI was used. Six mice per group were included in this experiment.

For immunohistochemistry using the anti-CD3 antibody, we followed the procedures described previously [39], [40]. Briefly, frozen sections were dried at room temperature for 30 min and rehydrated for 10 min in 0.1 M PBS at pH 7.0. Endogenous peroxidase activities were blocked by 3% hydrogen peroxide for 10 min and then washed three times with PBS. The sections were then incubated in Superblock (Thermo Scientific, Rockford, IL) for 1 h at room temperature. Antibody against CD3 was incubated with the tissue sections for 2 h at room temperature. After three 5-min washes, tissue sections were incubated for 1 h with a secondary biotinylated antibody and then with prediluted streptavidin-HRP for 45 min. Immunoreactivity was detected using diaminobenzidine (DAB) as the chromogen. Controls for nonspecific binding were performed by excluding primary antibodies. Brightfield images were captured at 10×10 magnification using a Nikon DXM 1200C camera attached to a Nikon Eclipse 80i microscope. The populations of CD3-immunoreactive cells in both the epithelium and the lamina propria underneath taste tissues were quantitatively measured using Image-Pro Plus image analysis software (version 6.0, Media Cybernetics, Inc., Bethesda, MD). This software allows analysis of brown-stained areas in tissue sections (obtained after immunohistochemistry with DAB staining). On the computer screen, two masks were used: one defined a threshold to determine the total areas of the epithelium and the lamina propria in a measured section; the other was applied over the captured image based on the color range of DAB-reactive immune cells. The masked areas within the epithelium and the lamina propria were measured and summed, and the size for each region was calculated. The cell population was expressed as the ratio of the colored area (cells) to the total area of tissue region measured. Eight MRL/lpr mice and nine MRL/+/+ mice were used for this experiment.

Double immunostaining with anti-Ki67 and anti-KCNQ1 antibodies were performed as described previously [24]. Fresh-frozen sections were used for this experiment. Tissue sections were fixed on slides in cold acetone for 30 sec. The rabbit polyclonal anti-KCNQ1 antibody was paired with the Cy3-conjugated goat anti-rabbit secondary antibody. Mouse monoclonal anti-Ki67 antibody was labeled with Alexa 488 Zenon Mouse IgG Labeling Kit (Invitrogen) following the manufacturer's protocol. Ki67-labeled cells in the basal regions surrounding taste buds, defined by KCNQ1 immunostaining, were counted. The average number of Ki67-labeled cells per taste bud profile was calculated. Four mice per group and six circumvallate sections from each mouse were included in the analysis.

### Hematoxylin and eosin (H&E) staining and taste bud profile measurements

MRL/lpr and MRL/+/+ mice (5 per group) at about 4–6 months of age were used for this experiment. Tongues were processed for histological studies as described above. Four to six tissue sections containing taste papillae were washed three times with deionized water and submerged in Harris Modified Hematoxylin Stain Solution (Mercedes Medical) for 3 min. After two rinses with water, sections were treated with acid alcohol solution for 1 min, washed three times with water, and then treated with 0.3% NH_4_OH for 1 min. Sections were then washed with water, dipped in 80% ethanol, and submerged in 1% eosin Y stain (Mercedes Medical) for 30–60 s. After sequential washes with ethanol and xylene, sections were mounted and imaged using a Nikon Eclipse 80i microscope. We measured the areas of taste bud profiles using Image-Pro Plus software. Only the taste bud profiles with taste pores were included in the measurement, so that just the taste bud profiles from the middle planes of taste buds were measured. On average, about 89 taste bud profiles were measured from each mouse.

### BrdU pulse-chase experiments

Five doses of BrdU (20 mg/kg of body weight per dose) were given to 5-month-old MRL/+/+ and MRL/lpr mice by intraperitoneal (i.p.) injections within a 12-h period, with interinjection intervals of 3 h. Mice were sacrificed at 1 and 5 days after the first BrdU injection. Four mice per group per time point were used. Tongues were fixed in 4% PFA/PBS solution for 1 h on ice and transferred to 20% sucrose/PBS solution for an overnight incubation at 4°C. Tissues were then embedded and cryosectioned into 10-µm-thick serial sections of circumvallate papillae.

Immunostaining with anti-BrdU and anti-KCNQ1 antibodies was performed as described previously [24]. Briefly, representative circumvallate sections (6 sections out of 26–28 serial sections per mouse) were selected from the top to the bottom of each circumvallate papilla (only the taste-bud-containing portion). The sections were separated from each other by about 40 µm to avoid resampling of the same taste buds. The positions of these sections along the circumvallate trench were approximately the same for all mice. Sections were washed twice in PBS solution containing 0.3% Triton X-100, followed by rinses with deionized H2O and then incubation in 4 N HCl for 20 min. After a second round of washes with PBS containing 0.3% Triton X-100 (pH 7.4), the sections were incubated with the blocking buffer at 4°C overnight. The anti-BrdU mouse monoclonal antibody was labeled with the Alexa 488 Zenon Mouse Antibody Labeling Kit following the manufacturer's recommended protocol. The freshly labeled antibody was added to the slides and incubated at room temperature for 2 h. The sections were washed twice with PBS containing 0.3% Triton X-100 solution and once with PBS (pH 7.4) and then postfixed in freshly prepared 4% PFA/PBS for 15 min at room temperature. The slides were washed three more times and blocked with the blocking buffer at room temperature for 1 h, and then incubated with rabbit anti-KCNQ1 antibody at 4°C overnight. A Cy3-conjugated goat anti-rabbit secondary antibody was added to sections. After 40-min incubation, sections were washed and mounted for imaging as described above.

BrdU-labeled cells in the circumvallate epithelium were classified as either perigemmal (nontaste epithelial) or intragemmal (taste) cells, with taste bud profiles defined by KCNQ1 immunostaining [24]. We counted the number of BrdU-labeled taste cells and divided it by the number of taste bud profiles to generate the average number of BrdU-labeled taste cells per taste bud profile. We also counted the number of BrdU-labeled perigemmal cells, as well as the total number of BrdU-labeled cells in the circumvallate epithelium. These numbers were divided by the area of the circumvallate epithelium to generate the number of BrdU-labeled perigemmal cells and the total number of BrdU-labeled cells per square millimeter (mm^2^) of circumvallate epithelium.

### Electrophysiological recordings of taste responses from the chorda tympani (CT) and the glossopharyngeal (GL) nerves

The procedures for CT and GL nerve dissection and recording have been described previously [41], [42], [43], [44]. Briefly, under pentobarbital anesthesia (50–60 mg/kg of body weight, i.p.), the trachea of each animal was cannulated, and the mouse was then fixed in the supine position with a head holder. For recordings from the CT nerve, the right CT nerve was exposed at its exit from the lingual nerve and cut near its entrance to the bulla. For recordings from the GL nerve, the right GL nerve was exposed by removal of the digastric muscle and posterior horn of the hyoid bone and then dissected free from underlying tissues and cut near its entrance to the posterior lacerated foramen. For whole-nerve recording, the entire nerve was placed on a platinum wire recording electrode. An indifferent electrode was positioned nearby in the wound. Neural responses resulting from chemical stimulations of the tongue were fed into an amplifier (Grass Instruments, West Warwick, RI), monitored on an oscilloscope and an audio monitor. Whole-nerve responses were integrated with a time constant of 1.0 s and recorded using a computer for later analysis using a PowerLab system (PowerLab/sp4; AD Instruments, Colorado Springs, CO).

For chemical stimulation, the tongue was enclosed in a flow chamber, and solutions were delivered into the chamber by gravity flow. The following solutions were used as stimuli: 0.1–20 mM quinine hydrochloride (QHCl), 0.01–1 M NaCl, 0.3–10 mM HCl, 1–30 mM citric acid, 0.01–1 M sucrose, 0.5–20 mM saccharin, 10–300 mM monosodium glutamate (MSG), 0.1 M monopotassium glutamate (MPG), 0.5–10 mM inosine-5′-monophosphate (IMP), and 0.1M NH_4_Cl. These concentrations of taste solutions were chosen because they evoke CT nerve responses ranging from weak to strong. These chemicals were dissolved in distilled water and used at ∼24°C. During recordings the test solutions were flowed for 30 s (CT recordings) or 60 s (GL recordings) at the same flow rate and temperature as distilled water used for rinsing the tongue (∼0.1 ml/s). The tongue was rinsed with distilled water for 1 min between successive stimulations. Eight to twelve MRL/+/+ and seven to ten MRL/lpr mice were recorded for CT nerve responses to taste solutions and 0.1M NH_4_Cl. Three mice were used for GL nerve recordings.

To analyze whole-nerve responses to each stimulus, the magnitudes of integrated responses at different time points after stimulus onset (at 5, 10, 15, 20, and 25 s for CT recordings, and at 10, 15, 20, 25, 30, 35, 40, 45, and 50 s for GL recordings) were measured and averaged. The relative response magnitude for each test stimulus was calculated against the response magnitude to 0.1 M NH_4_Cl, and this value was used for statistical analysis and for plotting dose-response curves.

### Brief-access tests

We performed brief access tests with 5- to 6-month-old MRL/lpr mice and MRL/+/+ control mice (7 MRL/lpr mice and 8 MRL/+/+ mice) using the Davis MS-160 mouse gustometer (Dilog Instruments, Tallahassee, FL). We followed standard procedures developed by several laboratories for testing mice [45], [46], [47], [48], [49], [50]. Briefly, mice were water-deprived for 22.5 h before 30 min training sessions and before test sessions for bitter, salty, and sour taste compounds. Water deprivation was used to motivate mice to sample these taste solutions. However, under these conditions mice are strongly motivated to lick, which would create a “ceiling effect" for testing palatable stimuli (i.e., in water-deprived mice, water licking rates are so high that adding a palatable substance does not further increase licking rate). We therefore used food and water-restriction (1.5 ml of water and 1 g of food) for 23.5 h before the test sessions for sweet and umami taste compounds, which is a standard procedure used in many studies [45], [46], [47], [48], [49], [50]. Mice were monitored for the loss of body weight during water and/or food restriction procedures. Those that lost more than 20% of their body weights were removed from the experiment. In each test session, mice were tested with a range of concentrations of taste compounds, along with one or two water controls. Each taste stimulus was presented for 5 s. Water and taste compounds were randomly presented to mice following random presentation schemes generated by the computer software. The following taste compounds were tested: QHCl (0.01, 0.03, 0.1, 0.3, 1, 3, and 10 mM), sucrose (0.03, 0.1, 0.2, 0.3, 0.6, and 1 M), IMP (0.3, 1, 3, 10, and 30 mM), NaCl (0.01, 0.03, 0.1, 0.2, 0.3, and 0.6 M), and citric acid (1, 3, 10, 30, 100, and 150 mM). We chose stimulus concentrations optimal for lickometer tests based on our previous data. The goal was to examine concentrations that evoke responses ranging from weak to strong. Strength of response is somewhat different in behavioral and electrophysiological tests; hence, different ranges of concentrations were sometimes used. Taste stimulus to water lick ratios were calculated by dividing the number of licks for taste compounds by the number of licks for water presented in the same test session.

### Statistical analyses

The numbers of animals used for different experiments are described above and in the figure legends. Calculations for quantitative data were performed in Microsoft Excel, except for electrophysiological recording data and behavioral test data, which were performed using the statistical analysis software packages Statcel (OMS, Tokyo, Japan) or Statistica (StatSoft, Inc., Tulsa, OK). Averaged data are presented in graphs as mean ± SEM. Student's *t* tests were performed for analyzing qPCR, histology, and immunostaining data to compare means between MRL/+/+ and MRL/lpr groups. Two-way ANOVA tests were performed for analyzing behavioral and nerve recording data using strain as a between-group factor and solution concentration as a within-group factor. *Post hoc t* tests or Fisher LSD tests were then performed to compare differences between mean responses of MRL/+/+ and MRL/lpr mice at different concentrations. *p*-Values<0.05 were considered significant.

## Results

### Taste tissues of MRL/lpr mice display characteristics of inflammation

**Figure 1:**
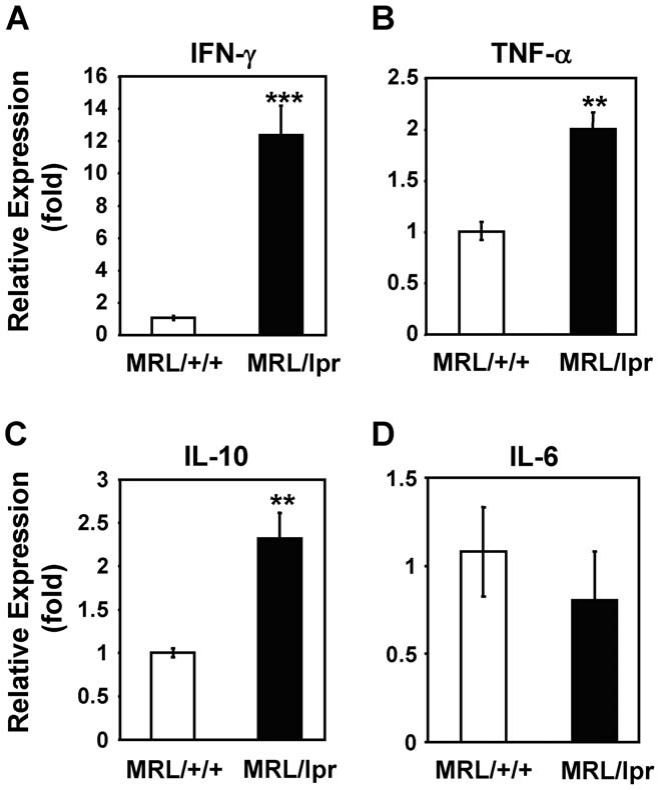
mRNA levels of IFN-γ, TNF-α, and IL-10 are elevated in the taste epithelium of MRL/lpr mice. qRT-PCR analysis of mRNA expression of IFN-γ (**A**), TNF-α (**B**), IL-10 (**C**), and IL-6 (**D**) in the circumvallate- and foliate-containing lingual epithelia of MRL/lpr and MRL/+/+ mice. Gene expression is shown as relative fold levels (mean ± SEM), with the mRNA level of each analyzed gene in MRL/+/+ mice arbitrarily set to 1. β-Actin was used as the endogenous control gene for relative quantification. For each mouse group, taste epithelial tissues from 3–4 mice were pooled for each set of sample preparations. Three independent sets of samples were prepared. Together, 9–12 mice per group were included in this study. Each qPCR reaction was run either in duplicate or triplicate. Student's *t* tests were performed. ** *p*<0.01; *** *p*<0.001.

To study whether systemic autoimmunity in MRL/lpr mice elevates inflammatory responses in taste tissues, we first analyzed mRNA levels of several inflammation-associated cytokines in the circumvallate and foliate epithelium by qRT-PCR analysis. As shown in Figure 1, the mRNA level of IFN-γ was markedly elevated (12.4-fold) in the taste epithelium of MRL/lpr mice compared with MRL/+/+ mice. mRNA levels of TNF-α and interleukin-10 (IL-10) were modestly but significantly higher (2.0- and 2.3-fold, respectively) in MRL/lpr mice than in control mice. In contrast, the mRNA level of IL-6 did not significantly differ in the taste epithelium of MRL/lpr and MRL/+/+ mice. Increased level of IFN-γ was also observed in nontaste tongue epithelium of MRL/lpr mice, although the level was considerably lower (about 5-fold lower) than in the taste epithelium of these mice (data not shown). The augmented mRNA levels of IFN-γ, TNF-α, and IL-10 in the taste tissues of MRL/lpr mice indicate the presence of elevated inflammatory responses in these tissues.

**Figure 2:**
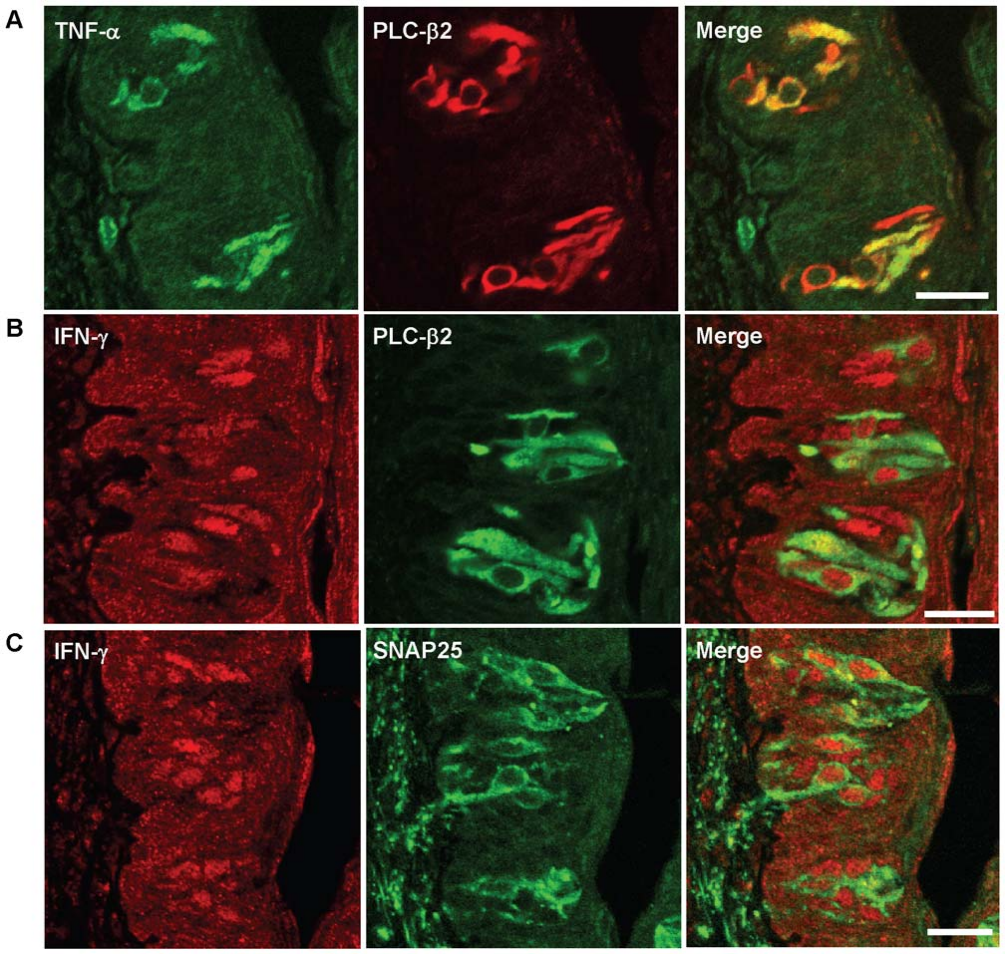
TNF-α and IFN-γ are expressed in subsets of taste bud cells. Confocal images of immunofluorescent staining of circumvallate papillae of MRL/lpr mice. (**A**) TNF-α is expressed in a subset of PLC-β2-positive cells. Immunoreactivities of TNF-α (green) and PLC-β2 (red) are colocalized in circumvallate taste buds of MRL/lpr mice. (**B** and **C**) IFN-γ is expressed in subsets of PLC-β2- or SNAP25-positive cells. Immunoreactivities of IFN-γ (red) are partially colocalized with PLC-β2 (green) and SNAP25 (green) in circumvallate taste buds of MRL/lpr mice. Scale bars, 20 µm.

Next, we examined what types of cells in the taste epithelium of MRL/lpr mice express TNF-α or IFN-γ by immunocolocalization using antibodies against PLC-β2 and SNAP25, markers for type II and III taste cells, respectively. As shown in Figure 2A, TNF-α immunoreactivity was predominantly localized to a subset of PLC-β2-positive cells. We rarely observed any TNF-α-immunoreactive cells without positive staining for PLC-β2 antibody in taste buds of MRL/lpr mice. On the other hand, IFN-γ-immunoreactive cells overlapped with PLC-β2- or SNAP25-positive cells (Fig. 2B–C). The immunoreactive signal for IFN-γ appeared to be perinucleus. These results suggest that TNF-α is preferentially expressed in a subset of type II cells, whereas IFN-γ is expressed in subsets of type II and III cells. These results are similar to our previously published data that showed the colocalization of TNF-α and IFN-γ with TrpM5 (transient receptor potential cation channel M5), another type II taste cell marker, in taste buds of C57BL/6 mice treated with LPS [24], although in MRL/lpr mice some IFN-γ-positive cells were SNAP25 positive (Fig. 2C).

**Figure 3:**
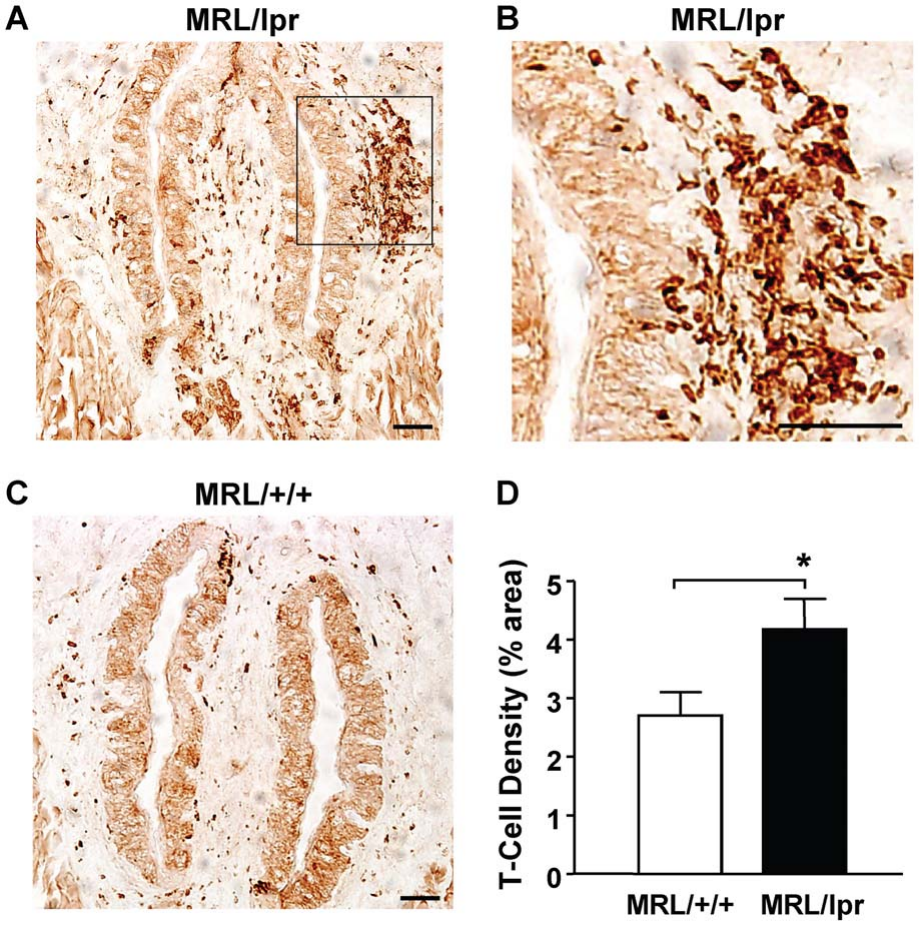
T-cell infiltration significantly increases in the circumvallate papilla and epithelium of MRL/lpr mice. Immunohistochemistry was performed using an antibody against CD3, a T-cell marker. (**A–C**) Images of the circumvallate papillae from MRL/lpr (**A** and **B**) and MRL/+/+ mice (**C**). T cells, either clustered or dispersed, are stained dark brown. The boxed region in **A**, which contains a large cluster of T cells in close proximity to the circumvallate epithelium, is shown at higher magnification in **B**. Scale bars, 40 µm. (**D**) T-cell densities (mean ± SEM) as percentage of anti-CD3-stained area against the total area of the observed circumvallate sections from MRL/+/+ and MRL/lpr mice. Eight MRL/lpr and nine MRL/+/+ mice were included in this study. Student's *t* tests were used for analysis. * *p*<0.05.

Consistent with elevated levels of inflammatory cytokines in the taste tissues of MRL/lpr mice, the number of CD3^+^ T lymphocytes was significantly increased in the taste papillae and epithelium of these mice (Fig. 3). We frequently observed large clusters of infiltrating T cells accumulating close to the taste epithelium in MRL/lpr mice (Fig. 3B). T-cell infiltration was also increased in nontaste tongue epithelium and its adjacent connective tissue layer in MRL/lpr mice compared with control mice (Fig. S1). Infiltration and accumulation of immune cells have been observed in other organs of MRL/lpr mice, such as the kidney, lung, and skin, and contribute significantly to tissue damage [51], [52], [53]. Our results suggest that T cells also infiltrate the taste tissues of MRL/lpr mice and thus may contribute to taste tissue damage in these mice.

### Taste buds of MRL/lpr mice show structural defects

**Figure 4:**
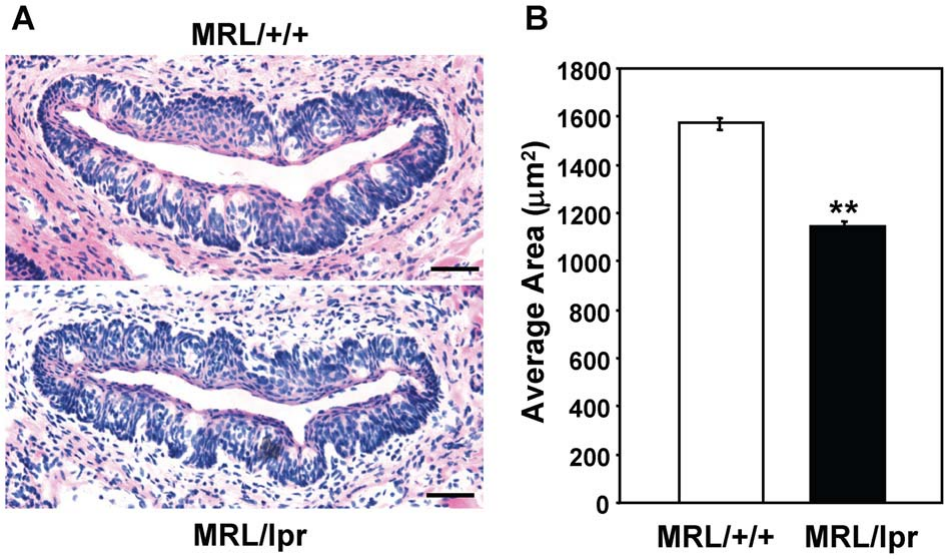
Taste buds of MRL/lpr mice are significantly smaller than those of MRL/+/+ mice. (**A**) H&E staining of circumvallate papillae of MRL/+/+ and MRL/lpr mice. The basal layer of the circumvallate epithelium is uneven, and taste buds are less organized in MRL/lpr mice. Scale bars, 40 µm. (**B**) Average areas of circumvallate taste bud profiles for MRL/+/+ and MRL/lpr mice. Five mice per group were included in the experiment. Areas of taste bud profiles were measured and averaged from at least four circumvallate sections per animal. Student's *t* tests were used for analysis. Data are means ± SEM. ** *p*<0.01.

To study whether long-term chronic inflammation in MRL/lpr mice affects taste bud structure, we examined circumvallate taste buds from MRL/lpr and MRL/+/+ mice at about 4–6 months of age. Sections containing circumvallate taste papillae were processed for H&E staining to reveal the gross structure of the taste epithelium. As shown in Figure 4A, the circumvallate taste epithelium of MRL/lpr mice had an irregular and disorganized appearance, and their taste buds were less recognizable than those of control mice. Quantitative analysis showed that taste buds of MRL/lpr mice were significantly smaller than those of MRL/+/+ mice (Fig. 4B). These results suggest that autoimmunity/chronic inflammation in MRL/lpr mice may affect the structural integrity of taste buds.

### Taste cell renewal is inhibited in MRL/lpr mice

In LPS-induced acute inflammation model, taste bud cell renewal is inhibited by inflammation [24]. To investigate if taste cell renewal is also affected by the chronic inflammation in MRL/lpr mice, we performed BrdU pulse-chase experiments in 4–6-month-old MRL/lpr and MRL/+/+ mice. In order to label an adequate number of taste cells, mice were injected with five doses of BrdU over a 12-h period. This injection scheme was shown previously to give robust labeling in the taste epithelium [24]. Taste tissues were collected at 1 and 5 days after the initial BrdU administration. Circumvallate sections from MRL/+/+ and MRL/lpr mice were immunostained with antibodies against BrdU and KCNQ1, a taste bud cell marker [36]. To follow the distribution of BrdU-labeled cells in the circumvallate epithelium, we counted the numbers of BrdU-labeled cells in taste buds and in perigemmal regions surrounding taste buds.

**Figure 5:**
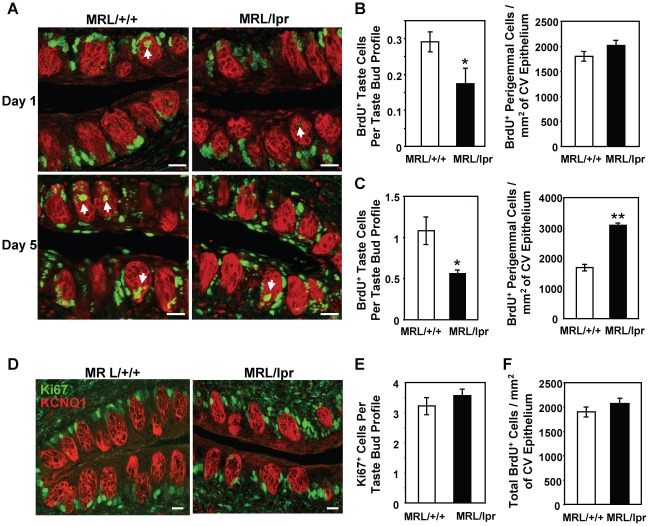
Taste bud cell renewal is inhibited in MRL/lpr mice. (**A**) BrdU (green) and KCNQ1 (red) double immunostaining of circumvallate papillae from MRL/+/+ and MRL/lpr mice injected with BrdU. Tissues were collected 1 and 5 days after BrdU injection (Day 1 and Day 5, respectively). Arrows denote some BrdU-labeled cells inside taste buds. (**B** and **C**) Quantitative analyses of BrdU-labeled cells in the circumvallate (CV) epithelium collected 1 day (**B**) and 5 days (**C**) after BrdU injection. Left, number of BrdU-positive taste cells per taste bud profile; right, number of BrdU-positive perigemmal cells per square millimeter (mm^2^) of circumvallate epithelium. (**D**) Ki67 (green) and KCNQ1 (red) double immunostaining: merged confocal fluorescent images of circumvallate papillae from MRL/+/+ and MRL/lpr mice. (**E**) Average number of Ki67-labeled cells in the basal region of taste bud profile. Ki67-labeled cells in the basal regions of circumvallate taste buds were counted, and the average numbers of Ki67-labeled cells per taste bud profile are shown for MRL/+/+ and MRL/lpr mice. (**F**) Total number of BrdU-positive cells (including cells in the perigemmal regions and inside the taste buds) per square millimeter (mm^2^) of circumvallate epithelium 1 day after BrdU injection. Four mice per group per time point were included in the experiments. Six circumvallate sections from each animal were included in the analyses. Data are means ± SEM. Student's *t* tests were used for statistical analysis. * *p*<0.05; ** *p*<0.01. Scale bars, 20 µm.

As shown in Figure 5A (upper panels), one day after BrdU injection, most BrdU-labeled cells were in the basal regions surrounding circumvallate taste buds, and only a small percentage of labeled cells entered the taste buds of MRL/+/+ and MRL/lpr mice, consistent with observations in other mouse strains [24], [54]. However, the number of BrdU-labeled cells that entered the taste buds of MRL/lpr mice was significantly reduced compared with that of MRL/+/+ mice (Fig. 5B, left). Five days after BrdU injection, although more BrdU-labeled cells entered the taste buds of both MRL/lpr and MRL/+/+ mice, the taste buds of MRL/lpr mice still contained significantly fewer labeled cells than those of MRL/+/+ mice (Fig. 5A, lower panels and 5C, left), suggesting that taste bud cell renewal is inhibited in MRL/lpr mice.

In contrast, cell renewal in the perigemmal regions of the circumvallate epithelium was not inhibited in MRL/lpr mice. One day after BrdU injection, the number of BrdU-labeled cells in the perigemmal regions of MRL/lpr mice was not significantly different from that of MRL/+/+ mice (Fig. 5B, right). Surprisingly, five days after BrdU injection, the number of BrdU-labeled perigemmal cells in MRL/lpr mice was significantly higher than that in MRL/+/+ mice (Fig. 5C, right), suggesting that more BrdU-labeled cells accumulate in the perigemmal regions of MRL/lpr mice. It should be noted that, although no significant strain difference was observed in the number of BrdU-labeled perigemmal cells one day after BrdU injection (Fig. 5B, right), this number from MRL/lpr mice was slightly higher than that from MRL/+/+ mice. It is possible that, at this time point, the strain difference was too small to be statistically significant.

In LPS-induced acute inflammation model, inhibition of taste cell renewal is primarily through suppression of progenitor cell proliferation. Recent studies suggest that taste bud cells and surrounding perigemmal keratinocytes are derived from a common progenitor cell population residing in the basal regions surrounding taste buds [55], [56]. These cells express cell proliferation markers such as Ki67. LPS treatment markedly reduces the number of Ki67-positive progenitor cells in the circumvallate epithelium [24]. To investigate whether the progenitor cell population in the circumvallate epithelium is affected in MRL/lpr mice, we performed immunostaining using anti-Ki67 antibody. We counted Ki67-positive cells in the basal regions surrounding circumvallate taste buds using KCNQ1 immunostaining as the taste bud marker. Our results show that MRL/lpr and MRL/+/+ mice have similar numbers of Ki67-positive cells in this niche (Fig. 5D–E), suggesting that the size of the progenitor cell population in the circumvallate epithelium of MRL/lpr mice is comparable to that of MRL/+/+ mice.

To investigate whether taste progenitor cell proliferation is inhibited in MRL/lpr mice, we counted the total number of BrdU-labeled cells (including both perigemmal and taste bud cells) per square millimeter (mm^2^) of circumvallate epithelium 1 day after BrdU injection. This number represents the total number of newborn cells in per square millimeter (mm^2^) of the circumvallate epithelium. Our results show that the total number of BrdU-labeled circumvallate epithelial cells is similar in MRL/lpr and MRL/+/+ mice (Fig. 5F), suggesting that the progenitor cells in the circumvallate epithelium proliferate at similar rates in the two mouse strains. Together, these data suggest that there is an inhibition of taste cell renewal in MRL/lpr mice. This inhibition is not due to reduced progenitor cell proliferation, but likely due to fewer newborn cells entering the taste buds.

### mRNA levels of gustducin, TrpM5, and NeuroD are reduced in the taste epithelium of MRL/lpr mice

**Figure 6:**
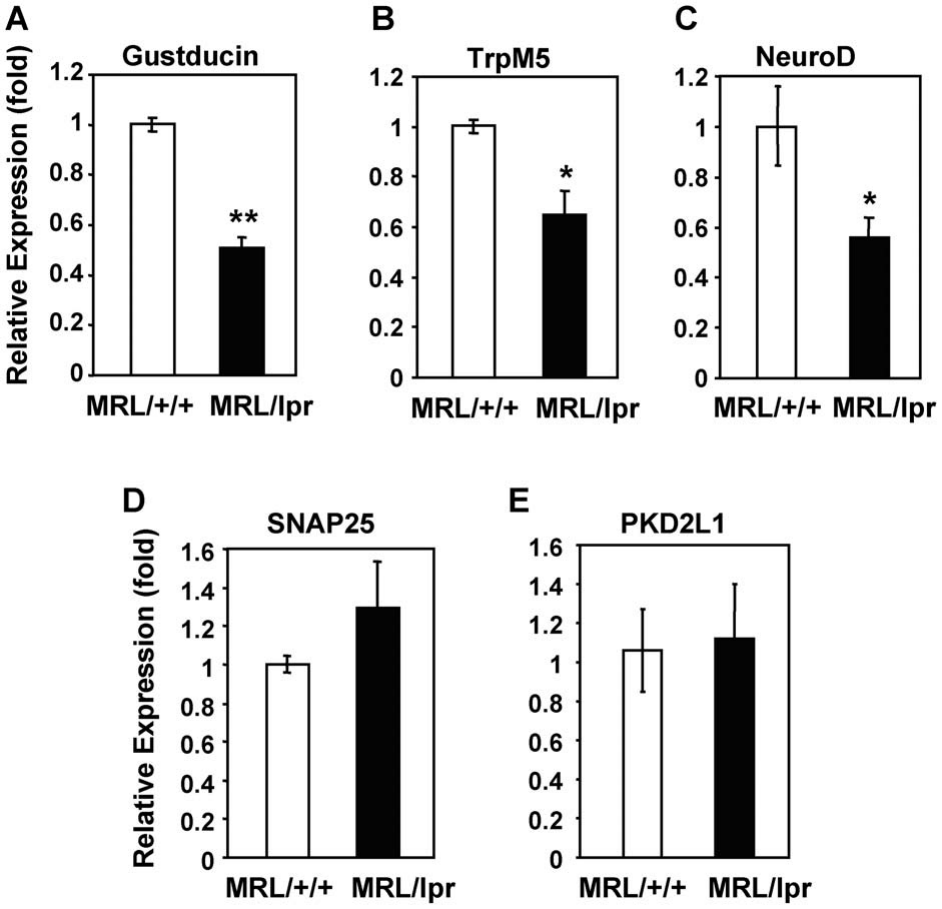
mRNA levels of gustducin, TrpM5, and NeuroD are reduced in the taste epithelium of MRL/lpr mice. qRT-PCR analysis was performed to quantify mRNA levels of gustducin (**A**), TrpM5 (**B**), NeuroD (**C**), SNAP25 (**D**), and PKD2L1 (**E**) in the circumvallate- and foliate-containing lingual epithelium prepared from MRL/+/+ and MRL/lpr mice. Gene expression (mean ± SEM) is shown in relative fold levels, with mRNA level of each analyzed gene in MRL/+/+ mice arbitrarily set to 1. β-Actin was used as the endogenous control gene for relative quantification. Epithelial tissues from 3–4 mice were pooled for each set of sample preparations. Three independent sets of samples were prepared. Each qPCR reaction was run either in duplicate or triplicate. Student's *t* tests were used for analysis. * *p*<0.05; ** *p*<0.01.

Because taste cell renewal is inhibited in MRL/lpr mice and their taste buds are smaller than those of control mice, next we investigated whether the expression of taste cell markers was affected. We performed qRT-PCR analyses to investigate mRNA expression of gustducin, TrpM5, NeuroD, SNAP25, and polycystic kidney disease 2-like 1 (PKD2L1) (Fig. 6). Among these markers, gustducin, TrpM5, and NeuroD are expressed in type II taste cells, and SNAP25 and PKD2L1 are expressed in type III taste cells. Gustducin and TrpM5 play important roles in the signal transduction of bitter, sweet, and umami tastes [57], [58], [59]. NeuroD may be involved in the differentiation of type II taste receptor cells [60]. SNAP25 is important for synaptic membrane fusion and is expressed in a subset of type III cells and in some nerve fibers in taste buds [61]. PKD2L1 is a marker for sour taste cells [62], [63], [64]. As shown in Figure 6A–C, mRNA levels of gustducin, TrpM5, and NeuroD were significantly reduced in the circumvallate and foliate epithelium of MRL/lpr mice compared with levels in control mice. Surprisingly, expression levels of SNAP25 and PKD2L1 did not significantly differ in the circumvallate and foliate epithelium of MRL/lpr versus MRL/+/+ mice (Fig. 6D–E). These results suggest that the mRNA levels of some type II cell-associated markers are selectively reduced in the taste buds of MRL/lpr mice.

### Taste buds of MRL/lpr mice contain fewer gustducin-positive taste receptor cells

**Figure 7:**
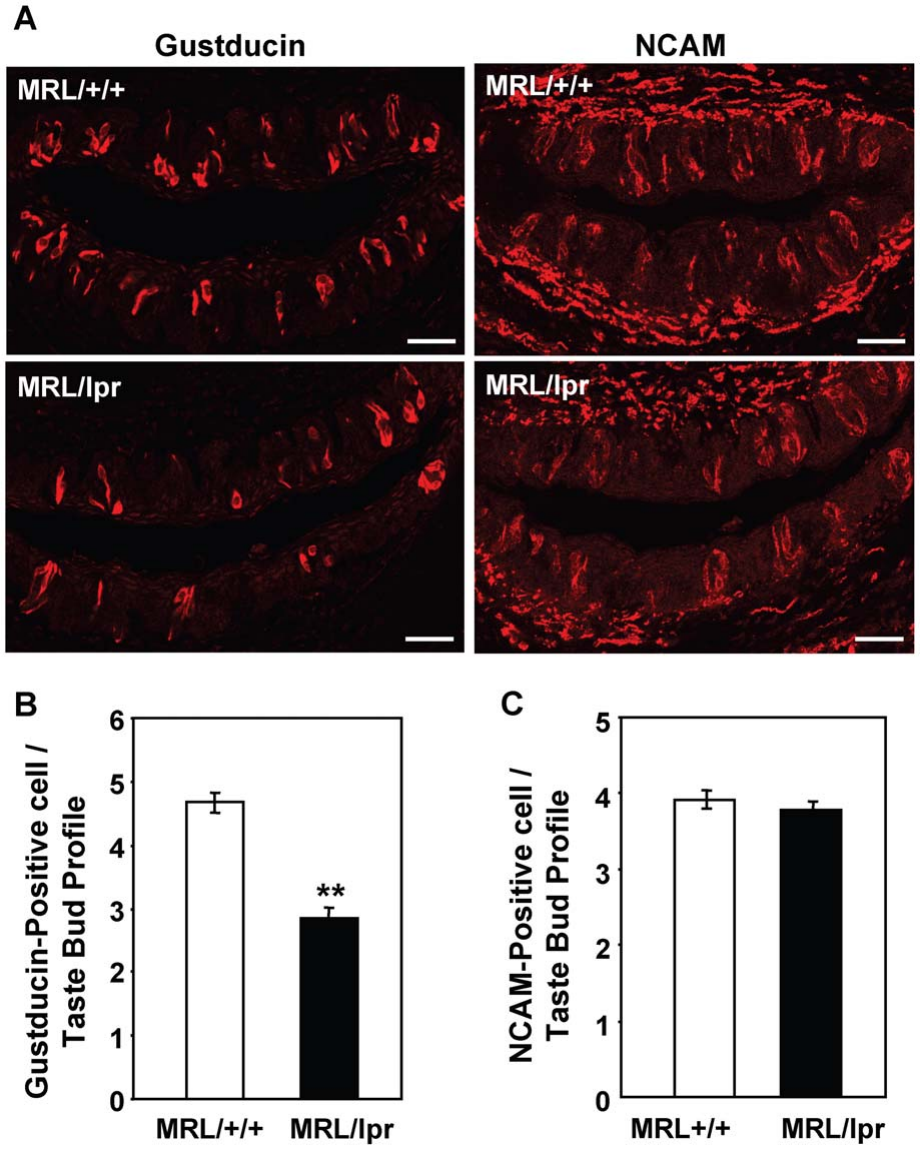
Number of gustducin-positive taste receptor cells is reduced in circumvallate taste buds of MRL/lpr mice. (**A**) Confocal images of immunofluorescent staining with antibodies against gustducin and NCAM. Circumvallate sections from MRL/+/+ and MRL/lpr mice were processed for immunostaining with anti-gustducin or anti-NCAM antibody as indicated. Scale bars, 40 µm. (**B** and **C**) Quantitative analyses of the average number of gustducin-positive (**B**) or NCAM-positive (**C**) cells per taste bud profile based on immunostaining data. Five mice per group were included in the experiment. Four circumvallate tissue sections per animal were included. Student's *t* tests were used. Data are means ± SEM. ** *p*<0.01.

To further investigate whether subsets of taste bud cells are preferentially affected in MRL/lpr mice, we performed immunohistochemical experiments using antibodies against gustducin, a type II taste cell marker, and NCAM or CA4, type III taste cell markers. As shown in Figure 7, circumvallate taste buds of 4- to 6-month-old MRL/lpr mice had significantly fewer gustducin-positive cells than those of control mice (Fig. 7A–B). Each circumvallate taste bud profile contained, on average, 4.7 gustducin-positive cells in MRL/+/+ mice and 2.8 gustducin-positive cells in MRL/lpr mice (we counted only the taste bud profiles with visible taste pores). In contrast, immunostaining with an antibody against NCAM showed no significant difference in the number of NCAM-positive cells per circumvallate taste bud profile in MRL/lpr mice versus controls (Fig. 7A and C). Similarly, fungiform taste buds from MRL/lpr mice also contained significantly fewer gustducin-positive cells than the buds from control mice, whereas no significant difference was observed in the number of CA4-positive type III cells per fungiform taste bud between MRL/lpr and control mice (Fig. S2). These results suggest that there is a selective reduction in the number of type II taste cells in MRL/lpr mice. These immunohistological observations are corroborated by the qRT-PCR analyses described above, demonstrating reduced mRNA levels of three type II cell markers, but not of type III cell markers in MRL/lpr mice (Fig. 6).

### MRL/lpr mice show decreased gustatory nerve responses to QHCl and saccharin

**Figure 8:**
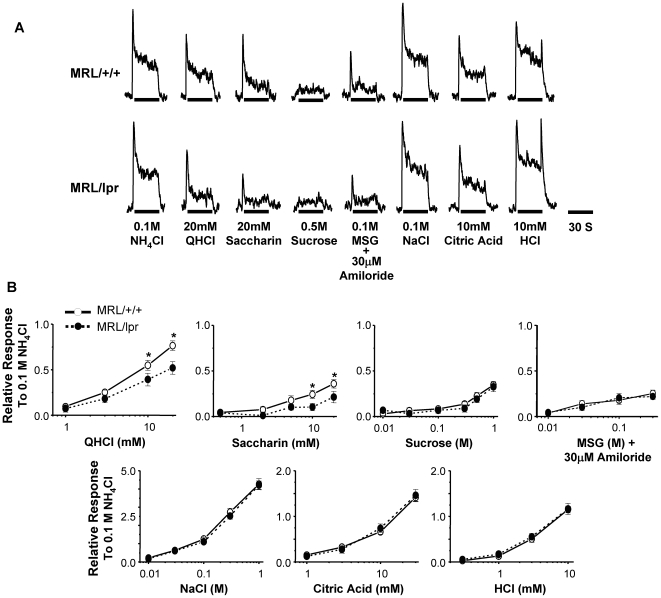
MRL/lpr mice show decreased CT nerve responses to quinine and saccharin. (**A**) Representative CT nerve responses to taste compounds. CT nerve responses to 0.1 M NH_4_Cl are shown as reference. (**B**) CT nerve responses (mean ± SEM) to several concentrations of QHCl, saccharin, sucrose, MSG, NaCl, citric acid, and HCl. Nerve responses to taste compounds were normalized against responses to 0.1 M NH_4_Cl. Nerve recordings were done with 8–12 MRL/+/+ mice and 7–10 MRL/lpr mice. Data were analyzed with two-way ANOVA with *post hoc* Fisher LSD tests. Responses to QHCl were significantly affected by strain (F_1,20_ = 4.7, *p* = 0.04) and strain×concentration interaction (F_3,60_ = 4.6, *p* = 0.006). Responses to saccharin were significantly affected by strain×concentration interaction (F_4,52_ = 2.7, *p* = 0.04). * *p*<0.05.

To examine peripheral gustatory nerve responses in MRL/lpr mice, we recorded responses of CT branch of cranial nerve VII to lingual applications of taste stimuli. Relative CT responses to taste stimuli were normalized against the response to 0.1 M NH_4_Cl. As shown in Figure 8, CT responses to QHCl (bitter) and saccharin (sweet) were significantly lower in MRL/lpr mice than in MRL/+/+ mice (two-way ANOVA tests). Responses to QHCl were significantly affected by strain (F_1,20_ = 4.7, *p* = 0.04) and strain×concentration interaction (F_3,60_ = 4.6, *p* = 0.006). Responses to saccharin were significantly affected by strain×concentration interaction (F_4,52_ = 2.7, *p* = 0.04). Although responses to sucrose (sweet) and MSG (umami) tended to be lower in MRL/lpr mice than in MRL/+/+ mice, the difference did not reach the level of significance (this is probably due to low responsiveness of the CT nerve to these compounds in both MRL/lpr and MRL/+/+ mice compared with some other strains used in previous studies) [42], [65]. CT responses to all tested concentrations of NaCl (salty) and to citric acid and HCl (sour) were similar in MRL/lpr and MRL/+/+ mice. We also recorded nerve responses to menthol, a transient receptor potential (TRP) channel ligand that activates somatosensory nerve fibers [66]. No significant difference in response to menthol was observed between MRL/lpr and MRL/+/+ mice (data not shown). Similar nerve response patterns were also observed in GL nerve recordings of MRL/lpr and MRL/+/+ mice (Fig. S3). GL nerve responses to QHCl and saccharin were consistently reduced in MRL/lpr mice compared with MRL/+/+ mice, whereas no difference was observed in GL responses to salty and sour compounds between the two mouse strains (Fig. S3). Together, the decreased nerve responses to QHCl and saccharin in MRL/lpr mice are consistent with the molecular and cellular data that show defects in type II taste receptor cells in these mice.

### MRL/lpr mice exhibit reduced behavioral responses to bitter, sweet, and umami taste compounds

**Figure 9:**
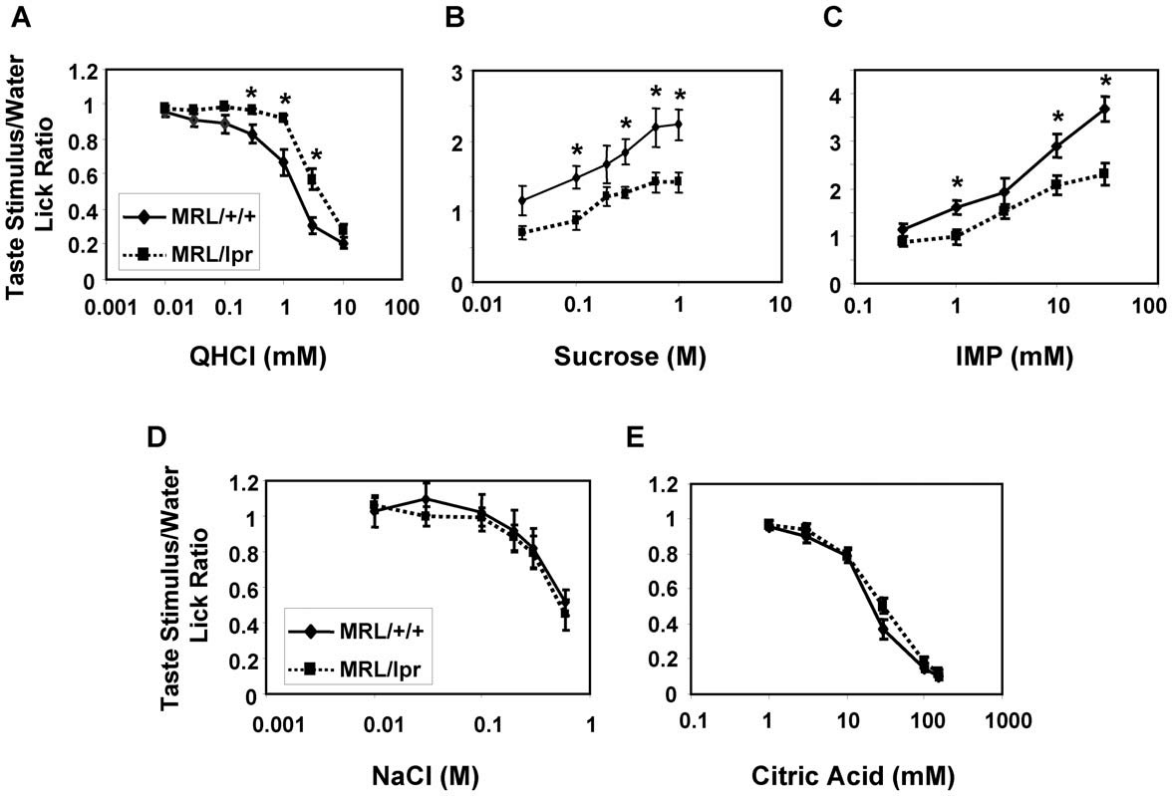
MRL/lpr mice exhibit reduced responses to bitter, sweet, and umami taste compounds in brief-access tests. Five-second brief-access tests were performed with MRL/lpr and MRL/+/+ mice to QHCl (**A**), sucrose (**B**), IMP (**C**), NaCl (**D**), and citric acid (**E**). Taste compounds and the tested concentrations are as indicated in the graphs. Lick ratios (mean ± SEM) were calculated as the number of licks of taste stimuli divided by the number of licks of water in each test session. N = 8 for MRL/+/+ mice; N = 7 for MRL/lpr mice. Data were analyzed with two-way ANOVA with *post hoc t* tests. * *p*<0.05.

MRL/lpr mice were previously reported to display reduced responsiveness to sucrose in consumption tests [67], [68]. Behavioral responses to other taste stimuli have not been examined in these mice. We performed brief access tests with 5- to 6-month-old MRL/lpr and MRL/+/+ mice. Our results showed that both MRL/lpr and MRL/+/+ mice displayed concentration-dependent changes in taste stimuli to water lick ratios: decreased ratios for QHCl, NaCl, and citric acid and increased ratios for sucrose and IMP, as taste solutions became more concentrated (Fig. 9). However, compared with controls, MRL/lpr mice exhibited significantly weaker licking suppression by QHCl and licking stimulation by sucrose and IMP at several concentrations (Fig. 9A–C). While MRL/+/+ mice had a decreased lick rate to 0.3 mM QHCl and increased lick rates to 0.1 M sucrose and 1 mM IMP relative to water, the lick rates to these stimuli in MRL/lpr mice were similar to their water lick rates (with taste stimuli to water lick ratio close to 1). These data suggest that MRL/lpr mice are less responsive to these taste stimuli than are control mice. Significant differences were also observed in responses to higher concentrations of QHCl, sucrose, and IMP between MRL/lpr and MRL/+/+ mice. It should be noted that no significant difference in licking responses to 10 mM QHCl was observed, which is most likely due to a “floor effect"; that is aversion at this concentration in both MRL/lpr and MRL/+/+ mice was so strong that it precluded any difference between these mice to be displayed. Reduced response to this concentration of QHCl was observed in MRL/lpr mice in taste nerve recordings (Fig. 8 and S3), which is not subject to the “floor effect". In contrast, MRL/+/+ and MRL/lpr mice had similar responses to NaCl and citric acid for all concentrations tested (Fig. 9D–E), suggesting selective alterations in bitter, sweet, and umami taste responses in MRL/lpr mice. These results are in line with results obtained from qPCR, immunohistochemistry, and nerve recording experiments (Figs. 6, 7, 8), suggesting that type II taste cells are selectively affected in MRL/lpr mice. However, these experiments do not rule out the possibility that the central nervous system or the gastrointestinal system may also contribute to the behavioral alterations we observed in MRL/lpr mice.

## Discussion

Taste abnormalities are associated with several forms of autoimmune diseases, among which Sjögren's syndrome is the best characterized. Studies have shown that Sjögren's syndrome patients exhibit reduced sensitivities to bitter, sour, sweet, and salty taste compounds [12], [13], [14]. Henkin et al. (1972) reported that 90% of Sjögren's syndrome patients in their study had bitter and sour detection and recognition thresholds elevated above the normal levels, whereas 72% and 38% of patients had salt and sweet thresholds above the normal levels, respectively [13]. A later study by Weiffenbach et al. (1995) also showed that the mean taste detection threshold for Sjögren's syndrome patients was approximately one-half log unit higher than that of controls. About 77% of patients had at least one taste threshold below the 10th percentile of controls, and among these patients 78% had low sensitivity for two or more taste qualities [12]. A more recent report by Gomez et al. (2004) also showed that Sjögren's syndrome patients were clearly hypogeusic for bitter and sour tastes and mildly hypogeusic for sweet and salty tastes [14]. Together, these human studies demonstrate that taste abnormalities can develop in this autoimmune disease.

Yet, the underlying causes of taste disorders in autoimmune diseases remain poorly understood. In Sjögren's syndrome patients, infiltration of lymphocytes often destroys normal function of the salivary glands and results in reduced production of saliva. Although early studies suggested an association between reduced saliva production and taste impairments in these patients [13], some later studies could not establish a direct correlation between saliva production and taste sensitivities [12], [14], suggesting that other factors may contribute to the taste abnormalities. Autoimmune diseases can also affect the central nervous system [10], [69], yet it remains unclear whether central nervous system symptoms affect taste perception.

Here we use a mouse model for human systemic lupus and Sjögren's syndrome to study the molecular and cellular basis of taste dysfunction. Our results show that, similar to human Sjögren's syndrome patients, MRL/lpr mice develop taste dysfunction. These mice show decreased nerve responses to the bitter substance QHCl and the sweet compound saccharin and reduced behavioral responses to bitter, sweet, and umami compounds. Furthermore, taste tissues in MRL/lpr mice show signs of chronic inflammation, characterized by increased levels of IFN-γ and TNF-α and infiltration of T cells into taste papillae. The taste buds of MRL/lpr mice are smaller and contain fewer gustducin-positive cells than those of controls, consistent with the reduced mRNA levels of gustducin, TrpM5, and NeuroD in the taste epithelium of these mice. BrdU pulse-chase experiment reveals that taste bud cell renewal is inhibited in MRL/lpr mice. Together, our results suggest that taste buds, especially type II taste receptor cells, are abnormal in this autoimmune/chronic inflammation model, which likely contributes to taste abnormalities observed in these mice and possibly in human systemic lupus and Sjögren's syndrome patients.

Unlike their neighboring stratified keratinized epithelial cells, taste receptor cells play important sensory functions and have to be readily accessible to the taste compounds in food and drink. Lacking a strong physical barrier, taste receptor cells are more vulnerable to harmful chemicals or pathogens in the oral environment. Inflammation is a defensive response to stress, toxins, injury, and infectious agents [70]. Perhaps it is not surprising that, compared with nontaste epithelial cells, taste bud cells express higher levels of multiple receptors, signaling molecules, and cytokines associated with inflammation [22], [23], [71]. It is possible that higher expression of these molecules ensures rapid and robust inflammatory responses when danger signals are detected.

However, chronic or excessive inflammatory responses are destructive and contribute to the development of a number of diseases [72], [73]. In taste papillae, elevated inflammatory responses have been observed in different experimental models. Intraperitoneal injection of LPS induces systemic inflammation as well as elevated local inflammatory response in the taste papillae. Levels of inflammatory cytokines, such as TNF-α, IFN-γ, IL-6, and IL-12, are highly up-regulated in the taste epithelium. This LPS-induced inflammation strongly inhibits taste progenitor cell proliferation and taste bud cell renewal and moderately shortens the average life span of taste bud cells [24]. In the gustatory nerve injury model, sectioning the CT nerve increased the number of neutrophils and macrophages in both the denervated and the intact sides of the tongue. Infiltration of neutrophils and macrophages can either impair the peripheral gustatory function or promote the recovery of taste function after CT nerve injury [74], [75], [76].

In this study, we show that in the MRL/lpr autoimmune disease model, levels of IFN-γ and TNF-α are augmented in the taste epithelium, which is accompanied by increased infiltration of T lymphocytes (Figs. 1, 2, 3), suggesting the presence of chronic inflammation in the taste tissue. We showed previously that injection of IFN-γ can elicit cell death in taste buds [22]. Yet, various apoptosis assays detected only a small increase in cell death in the taste buds of MRL/lpr mice (data not shown). On the other hand, BrdU pulse-chase experiments revealed that taste bud cell renewal in MRL/lpr mice is inhibited (Fig. 5). Interestingly, this experiment also shows that although there are fewer BrdU-labeled cells in the taste buds, there are more of them in the perigemmal regions in MRL/lpr mice, suggesting an abnormal distribution of BrdU-labeled cells between taste and nontaste lineages. Thus, it is possible that cell differentiation toward the taste lineages, particularly the type II cell lineage, was suppressed in MRL/lpr mice. However, further experiments are needed to test this hypothesis. Although recent studies suggest that a common progenitor cell population gives rise to both taste bud cells and perigemmal epithelial cells, it remains under debate whether type I, II, and III taste cells come from common or separate taste progenitor cells [77], [78], [79]. Many cytokines, including IFN-γ and TNF-α, can affect cell differentiation under some conditions [80], [81]. In the taste buds of MRL/lpr mice, a subset of type II cells produces TNF-α and subsets of types II and III cells produce IFN-γ (Fig. 2). These locally produced cytokines may affect taste cell differentiation and survival through autocrine and paracrine mechanisms depending on the expression of the cytokine receptors. Previously, we showed that the IFN-γ receptor is expressed in subsets of gustducin-positive (type II) and NCAM-positive (type III) cells [22]. TNF-α has two receptors, TNFR1 and TNFR2, which can mediate opposite effects depending on the presence of downstream signaling pathways [80]. For instance, activation of TNFR1 often induces cell death under various conditions, whereas activation of TNFR2 can promote cell survival [80]. Our preliminary studies suggest that TNFR1 and TNFR2 are expressed preferentially in type II and type III cells, respectively (data not shown). These expression patterns indicate that TNFR1 signaling or the combination of TNFR1 and IFN-γ receptor signaling may contribute to the loss of type II taste cells, whereas TNFR2 signaling may protect type III cells in MRL/lpr mice. The roles of these cytokines and their receptors in regulating taste cell death, survival, and differentiation will be further investigated in the future.

While taste cell renewal is inhibited in both the LPS-induced acute inflammation model and the MRL/lpr autoimmune/chronic inflammation model, the underlying mechanisms that lead to this inhibition appear to be different. In the LPS-induced acute inflammation model, inhibition of taste cell renewal is primarily due to suppression of progenitor cell proliferation, whereas in the MRL/lpr model, progenitor cell proliferation in the circumvallate epithelium is not suppressed. The reason for this difference in mechanisms is unclear. The cytokine expression profile in the taste epithelium of MRL/lpr mice is distinct from that of LPS-treated mice. The LPS-induced acute inflammatory response in the taste epithelium is dominated by TNF-α and IL-6 [24], whereas in the taste epithelium of MRL/lpr mice, IFN-γ is highly induced and TNF-α is modestly increased, and the level of IL-6 remains unchanged compared with controls (Fig. 1). Whether these differences in cytokine expression between the two models contribute to the divergence in the regulatory mechanisms will be investigated in future studies.

Type II taste receptor cells are preferentially affected in MRL/lpr mice. The number of gustducin-positive cells per taste bud is significantly reduced in these mice (Fig. 7 and S2). In contrast, the number of NCAM-positive cells per taste bud in MRL/lpr mice is comparable to that in controls. In addition, qRT-PCR analyses show that mRNA levels of the type II taste cells markers gustducin, TrpM5, and NeuroD are decreased in MRL/lpr mice, but not mRNA levels of the type III taste cell markers SNAP25 and PKD2L1 (Fig. 6). The reduction is about 50% in the number of gustducin-positive cells per taste bud, as well as in the mRNA levels of type II cell markers, suggesting that the decreased number of type II cells is the primary cause for the decline in the levels of type II cell markers. Type II taste receptor cells are essential for bitter, sweet, and umami taste reception and signaling [17], [82]. Sour and salty tastes, on the other hand, are likely detected by type III and/or I taste cells [21], [63], [64], [83], [84]. In line with these studies and in agreement with the observed type II cell defects, our nerve recording experiments show that MRL/lpr mice are less responsive to the bitter compound QHCl and the sweet substance saccharin, but their responses to salty and sour compounds are comparable to those of controls (Fig. 8 and S3). Behavioral experiments also show that MRL/lpr mice are less responsive to bitter, sweet, and umami compounds, but are normal in response to salty and sour compounds (Fig. 9). CT nerve responses to sucrose and MSG tended to be lower in MRL/lpr mice than in MRL/+/+ mice, but the difference did not reach the level of significance, probably due to low responsiveness of the CT nerve to these compounds in both MRL/lpr and MRL/+/+ mice compared with some other mouse strains [42], [65]. Sweet taste sensitivities vary among different inbred mouse strains, partially due to sequence variations in *Tas1r3* gene [85]. Whether polymorphisms in *Tas1r3* are responsible for the low nerve responses to sweet and umami compounds in these MRL mouse strains is currently under investigation. Nevertheless, our data from gene expression, histology, nerve recording, and behavioral tests all support the conclusion that type II taste receptor cells are selectively affected in MRL/lpr mice.

While the etiology may be complex, taste abnormalities are well recognized in patients with Sjögren's syndrome [2], [13]. In human psychophysical studies, Sjögren's syndrome patients show decreased sensitivities for sweet, bitter, salty, and sour taste compounds [12], [13], [14]. Taste abnormalities in systemic lupus patients, although previously recognized, are not well characterized. The autoimmune disease developed in MRL/lpr mice shares some characteristics with systemic lupus and Sjögren's syndrome. Yet, MRL/lpr mice did not differ significantly in their responses to salty and sour taste compounds in both behavioral and nerve recording experiments compared with controls. However, when comparing taste alterations between MRL/lpr model and Sjögren's syndrome patients, one has to consider that many Sjögren's syndrome patients also develop other illnesses and take various medications for treatment, which may complicate their taste abnormalities [13]. One of the advantages of using animal models to study the mechanisms of taste dysfunction is to minimize the effects of other factors. Although not without limitations, these models can provide valuable information for understanding the molecular and cellular bases of taste disorders associated with various diseases.

## Supporting Information

Figure S1 **T-cell infiltration in nontaste lingual epithelium.** (**A**) T-cell infiltration in nontaste lingual epithelium and the underlying connective tissue layer of MRL/lpr and MRL/+/+ mice: representative images from MRL/+/+ and MRL/lpr mice. Immunohistochemistry was performed using an anti-CD3 antibody. (**B**) A graph of T-cell densities as the percentage of anti-CD3-stained area against the total area of the observed tissue sections from MRL/+/+ and MRL/lpr mice. Student's *t* tests were used for analysis. Data are mean ± SEM. ** *p*<0.01.
(TIF)

Figure S2 **Reduced number of gustducin-positive taste cells in fungiform taste buds of MRL/lpr mice.** (**A**) Confocal images of immunofluorescent staining using antibodies against gustducin and CA4. Fungiform sections from MRL/+/+ and MRL/lpr mice were processed for immunostaining with anti-gustducin (Gust, red) and anti-CA4 (CA4, green) antibodies. DAPI (blue) was used to reveal all nuclei. (**B** and **C**) Quantitative analyses of the average number of gustducin-positive (**B**) or CA4-positive (**C**) cells per taste bud profile based on immunostaining data. Six mice per group were included in the experiment. Student's *t* tests were used. Data are mean ± SEM. * *p*<0.05.
(TIF)

Figure S3 **GL nerve responses to taste compounds.** (**A**) Representative GL nerve responses to various taste compounds for MRL/+/+ and MRL/lpr mice. GL nerve responses to 0.1 M NH_4_Cl are shown as reference. (**B**) Averaged GL nerve responses normalized against the responses to 0.1 M NH_4_Cl. The responses to QHCl and saccharin were reduced in MRL/lpr mice.
(TIF)
