# Supplementary figures and images for: Defects in the Peripheral Taste Structure and Function in the MRL/lpr Mouse Model of Autoimmune Disease
Source: PLoS One. 2012 Apr 19;7(4):e35588. doi: 10.1371/journal.pone.0035588 (PMC3334929; doi:10.1371/journal.pone.0035588)

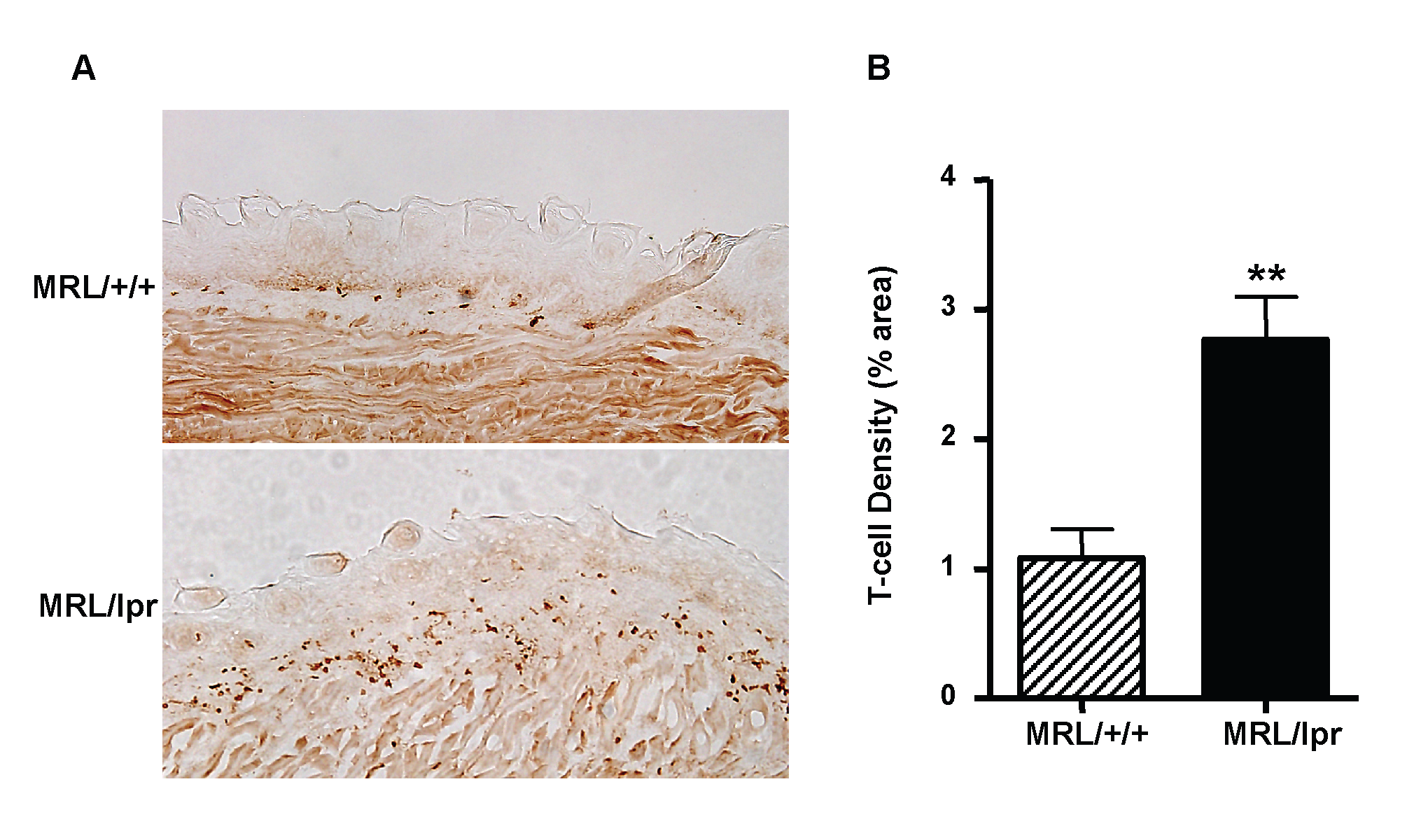

Supplement: Figure S1 — T-cell infiltration in nontaste lingual epithelium. (A) T-cell infiltration in nontaste lingual epithelium and the underlying connective tissue layer of MRL/lpr and MRL/+/+ mice: representative images from MRL/+/+ and MRL/lpr mice. Immunohistochemistry was performed using an anti-CD3 antibody. (B) A graph of T-cell densities as the percentage of anti-CD3-stained area against the total area of the observed tissue sections from MRL/+/+ and MRL/lpr mice. Student's t tests were used for analysis. Data are mean ± SEM. ** p<0.01. (TIF) [file pone.0035588.s001.tif]

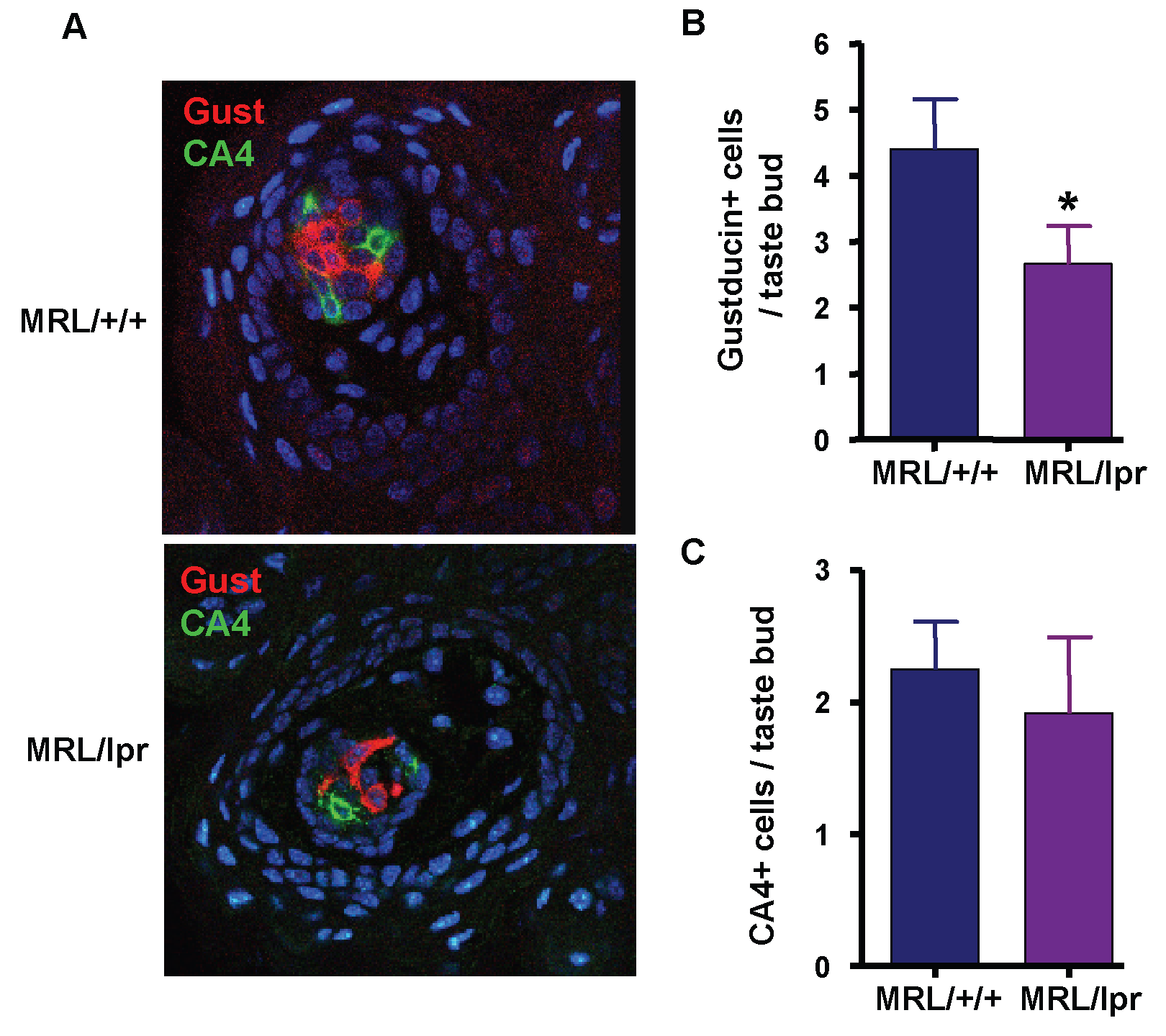

Supplement: Figure S2 — Reduced number of gustducin-positive taste cells in fungiform taste buds of MRL/lpr mice. (A) Confocal images of immunofluorescent staining using antibodies against gustducin and CA4. Fungiform sections from MRL/+/+ and MRL/lpr mice were processed for immunostaining with anti-gustducin (Gust, red) and anti-CA4 (CA4, green) antibodies. DAPI (blue) was used to reveal all nuclei. (B and C) Quantitative analyses of the average number of gustducin-positive (B) or CA4-positive (C) cells per taste bud profile based on immunostaining data. Six mice per group were included in the experiment. Student's t tests were used. Data are mean ± SEM. * p<0.05. (TIF) [file pone.0035588.s002.tif]

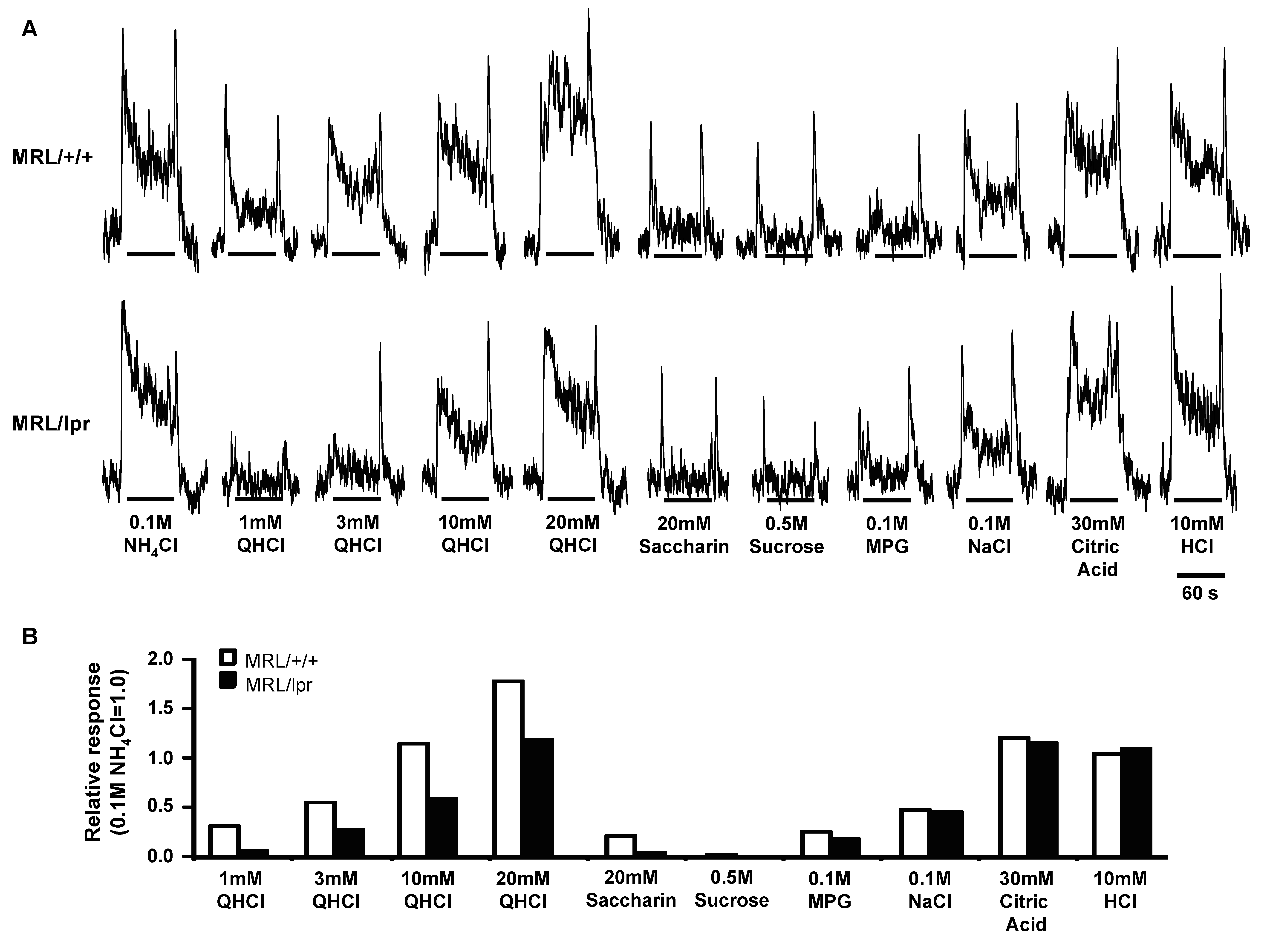

Supplement: Figure S3 — GL nerve responses to taste compounds. (A) Representative GL nerve responses to various taste compounds for MRL/+/+ and MRL/lpr mice. GL nerve responses to 0.1 M NH4Cl are shown as reference. (B) Averaged GL nerve responses normalized against the responses to 0.1 M NH4Cl. The responses to QHCl and saccharin were reduced in MRL/lpr mice. (TIF) [file pone.0035588.s003.tif]
